# Supplementary material for: Genetic comparisons of interleukin-17 reveal a framework for complex signaling evolution
Source: bioRxiv. 2026 Apr 14:2026.04.13.718218. Preprint. [Version 1] doi: 10.64898/2026.04.13.718218 (PMC13105070; doi:10.64898/2026.04.13.718218)
Supplement: Supplement 3 [file media-3.zip › Supplemental files for paper/ERC/IL17xIL17_comparisons/IL25_IL17C.pdf]

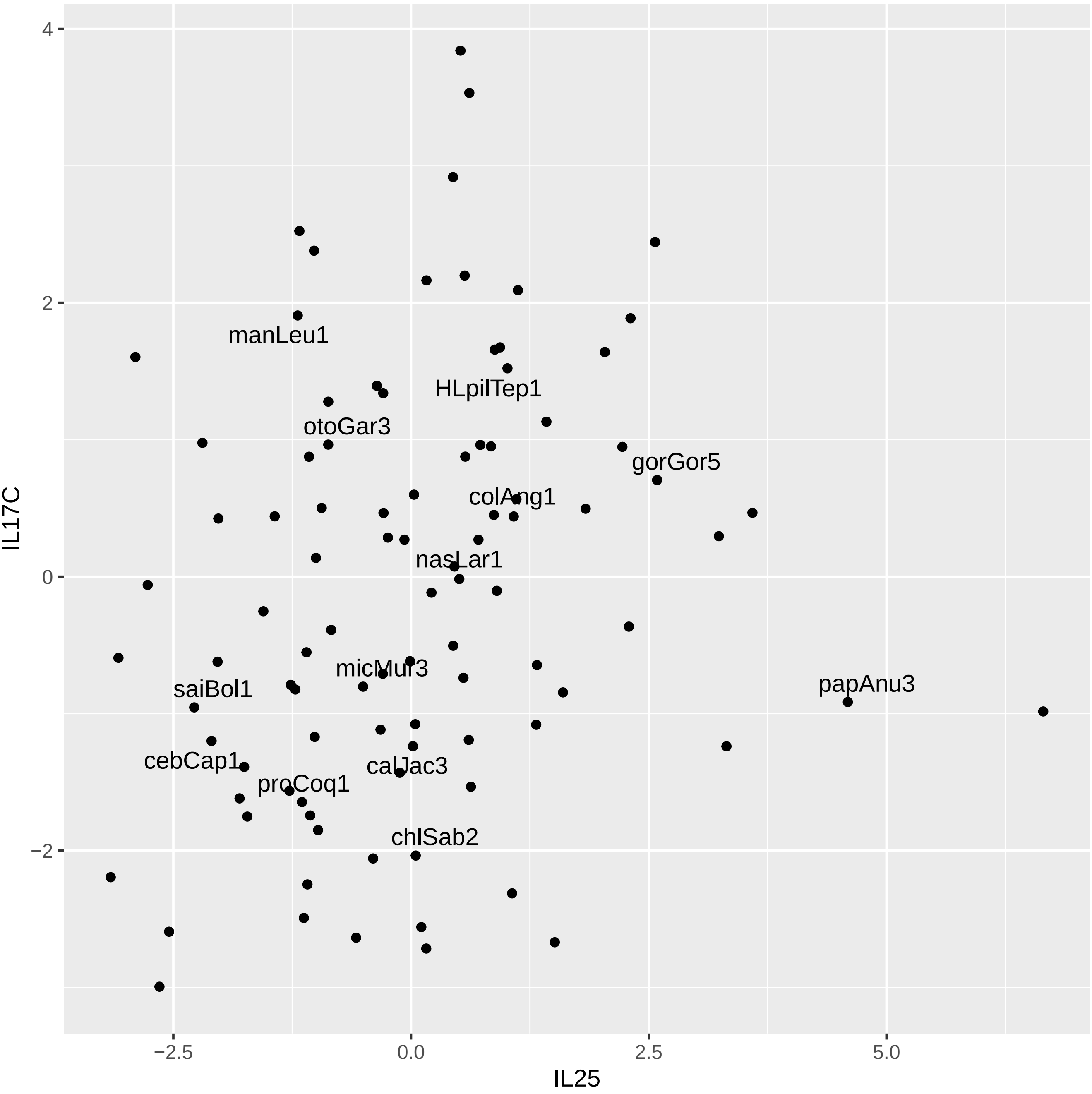

manLeu1
HLpilTep1
otoGar3
gorGor5
colAng1
nasLar1
micMur3
saiBol1
papAnu3
cebCap1
calJac3
proCoq1
chlSab2
IL17C
IL25
4
2
0
−2
−2.5
0.0
2.5
5.0
